# Predictors for surgical site infection after fasciotomy in patients with acute leg compartment syndrome

**DOI:** 10.1186/s13018-023-03589-9

**Published:** 2023-02-13

**Authors:** Shuo Yang, Yubin Long, Tao Wang, Junfei Guo, Zhiyong Hou

**Affiliations:** 1 Department of Orthopaedic Surgery, The Third Hospital of Hebei Medical University, Shijiazhuang, Hebei People’s Republic of China; 2 Orthopaedic Research Institute of Hebei Province, Shijiazhuang, 050051 Hebei People’s Republic of China; 3 The Third Department of Orthopedics, Baoding First Central Hospital, Baoding, Hebei People’s Republic of China; 4 NHC Key Laboratory of Intelligent Orthopaedic Equipment (The Third Hospital of Hebei Medical University), Shijiazhuang, People’s Republic of China

**Keywords:** Acute leg compartment syndrome, Fasciotomy, Open fractures, Body mass index, Smoking history

## Abstract

**Background:**

Surgical site infection (SSI) is one of the most common complications of orthopedic surgery, which can result in fever, pain, and even life-threatening sepsis. This study aimed to determine the predictors of SSI after fasciotomy in patients with acute leg compartment syndrome (ALCS).

**Methods:**

We collected information on 125 ALCS patients who underwent fasciotomy in two hospitals between November 2013 and January 2021. Patients with SSI were considered as the SSI group and those without SSI as the non-SSI group. Univariate analysis, logistic regression analysis, and receiver operating characteristic (ROC) curve analyses were used to evaluate patient demographics, comorbidities, and admission laboratory examinations.

**Results:**

In our research, the rate of SSI (26 of 125) was 20.8%. Several predictors of SSI were found using univariate analysis, including body mass index (BMI) (*p* = 0.001), patients with open fractures (*p* = 0.003), and patients with a history of smoking (*p* = 0.004). Besides, the levels of neutrophil (*p* = 0.022), glucose (*p* = 0.041), globulin (*p* = 0.010), and total carbon dioxide were higher in the SSI group than in the non-SSI group. According to the results of the logistic regression analysis, patients with open fractures (*p* = 0.023, OR 3.714), patients with a history of smoking (*p* = 0.010, OR 4.185), and patients with a higher BMI (*p* = 0.014, OR 1.209) were related predictors of SSI. Furthermore, ROC curve analysis indicated 24.69 kg/m^2^ as the cut-off value of BMI to predict SSI.

**Conclusions:**

Our results revealed open fractures, BMI, and smoking history as independent risk factors for SSI following fasciotomy in patients with ALCS and determined the cut-off value of BMI, enabling us to individualize the evaluation of the risk for SSI to implement early targeted treatments.

## Introduction

Acute compartment syndrome (ACS), commonly occurring after lower extremity fractures, is an orthopedic emergency caused by trauma or other factors that induce bleeding, swelling, or affect limb perfusion [1, 2]. Acute leg compartment syndrome (ALCS) is reported to affect 7.3 out of every 100,000 men and 0.7 out of every 100,000 women in the general population [3]. Previous studies have explained the pathological mechanisms of ALCS: Fluid transfers between the blood, extracellular and intracellular spaces, leading to increased tissue pressure inside the musculature [4]. The high pressure within the compartment reduces capillary blood flow and tissue pO_2_, eventually leading to muscle ischemia and necrosis. To the best of our knowledge, the diagnosis of ALCS is based on pressure measurements in the compartment as well as various clinical features known as the 5 Ps: pain out of proportion, paresthesias, pallor, paralysis, and pulselessness [5, 6]. However, delaying diagnosis or treatment may result in poor outcomes, such as permanent nerve and muscle damage, amputation, and even death [7]. Therefore, quick identification and subsequent immediate surgical fasciotomy are necessary to avoid the severe consequences associated with ALCS.

As the most efficient method for ALCS, fasciotomy has been demonstrated to significantly reduce complications for patients by reducing pressure and reestablishing blood flow to the impacted compartment [5, 8]. Fasciotomy, however, could also lead to several complications, such as bleeding, deep venous thrombosis (DVT), and muscle herniation [9–11], among which surgical site infection (SSI) is one of the most common complications, affecting 33% of surgical patients [12]. In a previous study, Morris et al. found that as many as 36% of tibial plateau fracture patients with ALCS who subsequently underwent fasciotomy suffered infections [13]. Recent research has also suggested that SSI rates could reach 30% [14, 15]. As a result, such infection can cause sepsis, reducing the viability of the injured limb and then leading to widespread organ failure [16], as well as 34.3% of all adverse events associated with surgery [17]. In addition, the infection has also been shown to be associated with readmission and prolonged lengths of stay [18, 19], ultimately lowering patients' quality of life significantly. Therefore, early identification of risk factors for SSI is beneficial for the prevention of these adverse outcomes.

Previous studies reported several risk factors for the development of SSI in patients with lower extremity fractures, such as cerebrovascular disease, heart disease, prolonged intra-operative duration, smoking, extended preoperative stay, allograft or bone substitute, elevated fasting blood glucose level, decreased albumin level, and abnormal NEUT count [20–22]. However, studies focusing on SSI after fasciotomy in patients with ALCS are scarce. Therefore, the aim of this study is to investigate the risk factors for SSI in ALCS patients who received fasciotomy.

## Materials and methods

### Ethics statement

Our study reviewed the electronic medical records of all ALCS patients diagnosed and treated in the 3rd Hospital of Hebei Medical University and Baoding No. 1 Central Hospital between November 2013 and January 2021. We obtained ethical permission from the institutional review boards of these two hospitals in compliance with the Helsinki Declaration's ethical criteria of 1964 (NCT04529330, S2020-022-1) (2022116).

### Patients

**Fig. 1:**
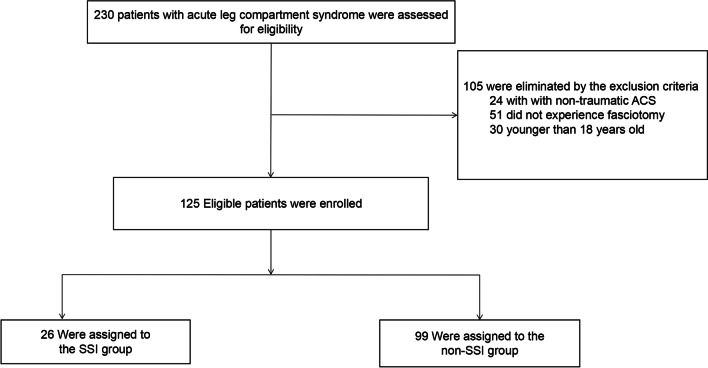
Flow diagram of included patients

This retrospective study was conducted at the 3rd Hospital of Hebei Medical University and Baoding No.1 Central Hospital, both of which were tertiary hospitals with a level I trauma center. Patients with traumatic ALCS and those with complete medical records could be included in this study. The exclusion criteria are: (1) patients younger than 18 years old; (2) patients with non-traumatic ALCS; and (3) patients who did not experience fasciotomy (Fig. 1).

According to the criteria above, we collected 125 patients (114 men and 11 women) with ALCS who had experienced fasciotomy. We divided these patients into the SSI group and the non-SSI group based on whether or not they developed SSI following fasciotomy. The diagnosis of ALCS is commonly controversial and is based on clinical evaluation and compartment pressure measurement [5]. Nevertheless, clinical diagnosis may be challenging because intrafascial pressure measurements were not frequently conducted in ALCS patients due to the severity of their condition. Therefore, the most reliable indicator of ACS is the change of color in muscle necrosis color during fasciotomy. In our study, the diagnosis of ALCS was made by two or more experienced orthopedic surgeons with at least ten years of clinical experience. Besides, based on the Center for Disease Control (CDC) 's definition of SSI [23], the depth of infection at the surgical site was traditionally categorized as superficial SSI, deep SSI, and SSI involving an organ. When performing fasciotomy, the fascial layer that traditionally separated the superficial and deep compartments was removed, allowing these two chambers to connect. The characteristics of this procedure dictate that it is nearly impossible to distinguish between superficial and deep infections. Therefore, all fasciotomy wound infections in this study were classified as deep infections [24]. Each patient who required fasciotomy would be given first- or second-generation cephalosporins as prophylaxis for infection during the procedure and for three days afterward. However, fasciotomy remained associated with a high postoperative infection incidence. In these patients, we could culture bacteria from purulent discharge and observe infection-specific phenomena such as odor, darkened granulation tissue, fever (*T* > 38 °C), swelling, pain involving deep soft tissues, and so on [25, 26]. The surgical incision situation and the bacterial culture results determined whether to perform the open surgical intervention of SSI. Multiple debridements may be required to contain the infection's progression.

This study collected patient demographics, comorbidities, and preoperative laboratory examinations. The demographics covered age, gender, BMI, fracture types, ASA score (American Society of Anesthesiologists score), smoking, alcohol, injury mechanism, time from injury to admission, time from injury to fasciotomy, and whether or not a dehydrating agent was used. The ASA score was classified into two groups: grades 1–2 and grades 3–4. Comorbidities include coronary heart disease, cerebral infarction, hypertension, diabetes, arrhythmia, and anemia. We also investigated many preoperative laboratory indicators, such as albumin (ALB), alkaline phosphatase (ALP), aspartate aminotransferase (AST), alanine transaminase (ALT), Calcium (Ca), K, Na, Mg, P, Cl, globulin (GLOB), cholinesterase (CHE), creatine kinase (CK), creatinine(CREA), direct bilirubin (DBIL), glucose (GLU), lactic dehydrogenase (LDH), triglyceride (TG), total cholesterol (TC), total carbon dioxide (TCO_2_), ureophil (UREA), uric acid (UA), basophil (BAS), eosinophil (EOS), hematocrit (HCT), hemoglobin (HGB), immature (IMM), lymphocyte (LYM), mean corpuscular hemoglobin concentration (MCHC), monocyte (MON), mean platelet volume (MPV), neutrophil (NEU), platelet (PLT), red blood cell (RBC), white blood cell (WBC).

### Statistical analysis

We used SPSS software (version 25.0, SPSS Inc., New York, USA) in this study and regarded *p* < 0.05 as statistically significant. The Shapiro–Wilk test was used to determine the normality of continuous variables. If normality was met, these variables were usually expressed as mean ± SD (standard deviation) using the t-test. Besides, the Mann–Whitney U test would be utilized in situations when the variables did not satisfy normality. For the classified variable, expressed as a number and its percentage, we used the Chi-square and Fisher's exact tests to compare the between-group difference. Furthermore, to identify independent risk factors for SSI after fasciotomy in patients with ALCS, we used binary logistic regression analysis to identify the best predictors of SSI.

Once the Youden index (sensitivity + specificity-1) reached its maximum, we commonly utilized ROC (receiver operating characteristic) analysis to identify the appropriate cut-off values for continuous variables, such as BMI. According to the cut-off value, these indices were divided into low-risk and high-risk groups. The diagnostic ability was determined by the area under the ROC curve (AUC), which varied from 0 to 100 percent, with a larger area indicating more diagnostic capability.

## Result

This research involved 125 patients, including 114 males and 11 females, all of whom had prior fasciotomy experience. The incidence of SSI after fasciotomy was 20.8%, with 26 patients affected and 99 unaffected.

Table 1 indicates that there were statistically significant differences between the SSI and non-SSI groups in terms of BMI (*p* = 0.001), smoking history (*p* = 0.004), and patients with open fractures (*p* = 0.001). This table showed that the BMI levels in the SSI group were higher than those in the non-SSI group. In addition, compared to the non-SSI group, patients in the SSI group were more likely to have a smoking history or suffer open fractures. But there were no significant differences between the two groups in terms of age, multiple fractures, mechanism of injury, time from injury to admission, time from injury to fasciotomy, ASA scores, alcohol history, and whether or not a dehydrating agent was used (all *p* > 0.05).

**Table 1:** Demographics data of patients with and without SSI

Characteristics	SSI group (*n* = 26)	Non-SSI group (*n* = 99)	*p*
Age, years	37.5 (24.8–48.0)	43.0 (28.0–55.0)	0.521
*Gender, n (%)*			0.540
Male	25 (96.2%)	89 (89.9%)	
Female	1 (3.8%)	10 (10.1%)	
BMI, kg/m^2^	26.2 (25.0–27.7)	24.3 (22.5–26.1)	0.001*
*Open fracture, n (%)*			0.003*
Yes	14 (53.8%)	24 (24.2%)	
No	12 (46.2%)	75 (75.8%)	
*Multiple fracture, n (%)*			0.208
Yes	8 (30.8%)	44 (44.4%)	
No	18 (69.2%)	55 (55.6%)	
*Mechanism of injury, n (%)*			0.907
Car crash injury	10 (38.5%)	31 (31.3%)	
Fall Injury	6 (23.1%)	23 (23.2%)	
Crush injury	4 (15.4%)	13 (13.1%)	
Hurt by a heavy object	3 (11.5%)	13 (13.1%)	
Unknown trauma	3 (11.5%)	19 (19.2%)	
Time from injury to admission, hours	5.0 (3.0–10.3)	6.0 (4.0–11.0)	0.360
< 12	22 (84.6%)	78 (78.8%)	0.234
12–24	2 (7.7%)	18 (18.2%)	
> 24	2 (7.7%)	3 (3.0%)	
Time from injury to fasciotomy, hours	12.0 (8.5–22.8)	10.0 (7.0–15.0)	0.110
≤ 12	14 (53.8%)	62 (62.6%)	0.339
12–24	6 (23.1%)	25 (25.3%)	
> 24	6 (23.1%)	12 (12.1%)	
*ASA classification, n * (*%)*			1.000
I, II	22 (84.6%)	85 (85.9%)	
III, IV	4 (15.4%)	14 (14.1%)	
*Smoking history, n * (*%)*			0.004*
Yes	11 (42.3%)	16 (16.2%)	
No	15 (57.7%)	83 (83.8%)	
Alcohol history, *n* (%)			0.938
Yes	5 (19.2%)	16 (16.2%)	
No	21 (80.8%)	83 (83.8%)	
*Whether to use dehydrating agent*			0.367
Yes	19 (73.1%)	63 (63.6%)	
No	7 (26.9%)	36 (36.4%)	

BMI, body mass index; ASA, American Society of Anesthesiologists; Values are presented as the number (%) or the median (interquartile range)

*p < 0.05, statistical significance

The findings of the comparison of the two groups' comorbidity data are shown in Table 2. However, we could not find any statistically significant differences between the two groups in terms of the existence or absence of the comorbidities that were investigated, including coronary heart disease, arrhythmia, hypertension, diabetes, cerebral infarction, anemia, and hypoproteinemia (all *p* > 0.05). Table 3 presents the findings of the laboratory tests performed on both the SSI and non-SSI groups. We found that the SSI group had significantly higher levels of NEU (*p* = 0.022), GLOB (*p* = 0.010), TCO_2_ (*p* = 0.033), and glucose (*p* = 0.041) than the non-SSI group. However, there were no significant differences in other laboratory data between these two groups (all *p* > 0.05).

**Table 2:** Comorbidities data of patients with and without SSI

	SSI group (*n* = 26)	Non-SSI group (*n* = 99)	*p*
*Arrhythmia, n (%)*			0.964
Yes	1 (3.8%)	4 (4.0%)	
No	25 (96.2%)	95 (96.0%)	
*Coronary heart disease, n * (*%)*			0.798
Yes	1 (3.8%)	5 (5.1%)	
No	25 (96.2%)	94 (94.9%)	
*Hypertension, n * (*%)*			0.872
Yes	4 (15.4%)	14 (14.1%)	
No	22 (84.6%)	85 (85.9%)	
*Diabetes, n * (*%)*			1.000
Yes	0 (0.0%)	3 (3.0%)	
No	26 (100.0%)	96 (97.0%)	
*Cerebral Infarction, n * (*%)*			0.833
Yes	1 (3.8%)	3 (3.0%)	
No	25 (96.2%)	96 (97.0%)	
*Anemia, n * (*%)*			0.624
Yes	10 (38.5%)	33 (33.3%)	
No	16 (61.5%)	66 (66.7%)	
*Hypoproteinemia, n * (*%)*			0.783
Yes	4 (15.4%)	20 (20.2%)	
No	22 (84.6%)	79 (79.8%)	

Values are presented as the number (%) or the median (interquartile range)

*p < 0.05, statistical significance

**Table 3:** Laboratory results of patients with and without SSI

	SSI group (*n* = 26)	Non-SSI group (*n* = 99)	*p*
BAS	0.05 (0.02–0.06)	0.05 (0.01–0.05)	0.842
E0S	0.13 (0.01–0.16)	0.13 (0.07–0.14)	0.773
HCT	36.05 (33.93–38.18)	36.17 (33.80–40.50)	0.253
HGB	121.64 (113.23–131.08)	122.12 (113.00–137.40)	0.280
IMM	0.18 (0.08–0.23)	0.18 (0.06–0.19)	0.867
LYM	1.54 (1.16–1.63)	1.62 (1.24–1.78)	0.154
MON	1.05 (0.74–1.11)	1.05 (0.71–1.07)	0.464
MPV	8.81 (8.22–9.58)	8.86 (8.18–9.10)	0.839
NEU	12.77 (9.86–17.71)	12.70 (9.27–13.77)	0.022*
PLT	201.13 (141.00–230.70)	200.82 (181.00–232.00)	0.961
RBC	4.42 (3.64–7.60)	4.48 (3.67–8.05)	0.774
WBC	15.42 (11.42–19.71)	15.50 (11.91–16.79)	0.843
ALB	34.79 (32.15–35.80)	34.28 (33.00–39.60)	0.756
ALP	70.63 (65.75–71.23)	73.00 (54.00–73.00)	0.932
ALT	51.82 (30.25–52.37)	50.00 (28.00–63.07)	0.723
AST	103.83 (33.25–103.83)	63.00 (30.00–143.06)	0.927
Ca	2.09 (2.09–2.18)	2.07 (1.99–2.21)	0.129
CHE	6.56 (5.86–6.95)	6.37 (5.47–6.80)	0.121
CK	3757.27 (421.40–3757.27)	1600.00 (389.25–5179.78)	0.765
CL	104.37 (100.84–104.48)	104.08 (102.80–106.20)	0.896
CREA	69.88 (69.16–74.60)	72.61 (60.76–72.62)	0.976
DBIL	5.25 (4.40–5.50)	5.23 (3.36–6.00)	0.121
GLOB	22.03 (22.02–24.78)	21.82 (19.20–22.40)	0.010*
GLU	8.00 (6.88–8.26)	7.95 (6.30–7.95)	0.041*
K	3.99 (3.94–4.15)	4.02 (3.68–4.12)	0.694
LDH	735.95 (420.25–765.97)	752.00 (276.14–935.50)	0.548
Mg	0.80 (0.79–0.89)	0.81 (0.76–0.85)	0.888
Na	137.78 (135.08–139.03)	137.28 (136.00–139.30)	0.534
P	1.14 (1.09–1.20)	1.17 (0.99–1.23)	0.621
TC	3.30 (2.81–3.31)	3.24 (2.75–3.66)	0.821
TCO_2_	23.42 (22.83–23.42)	23.53 (22.00–25.30)	0.033*
TG	1.37 (1.01–1.37)	1.38 (0.80–1.38)	0.347
TP	56.82 (54.00–63.47)	56.10 (52.00–61.50)	0.145
UA	325.83 (282.25–325.83)	332.35 (261.00–359.00)	0.286
UREA	5.33 (4.54–5.74)	5.68 (4.50–5.79)	0.292

BAS, basophil; EOS, eosinophil; HCT, hematocrit; HGB, hemoglobin; IMM, immature; LYM, lymphocyte; MCHC, mean corpuscular hemoglobin concentration; MON, monocyte; MPV, mean platelet volume; NEU, neutrophil; PLT, platelet; RBC, red blood cell; WBC, white blood cell; ALB, albumin; ALP, alkaline phosphatase; AST, aspartate aminotransferase; ALT, alanine transaminase; GLOB, globulin; CHE, cholinesterase; CK, creatine kinase; CREA, creatinine; DBIL, direct bilirubin; GLU, glucose; LDH, lactic dehydrogenase; TG, triglyceride; TC, total cholesterol; TCO_2_, total carbon dioxide; UREA, ureophil; UA, uric acid; Values are presented as the number (%) or the median (interquartile range)

*p < 0.05, statistical significance

**Fig. 2:**
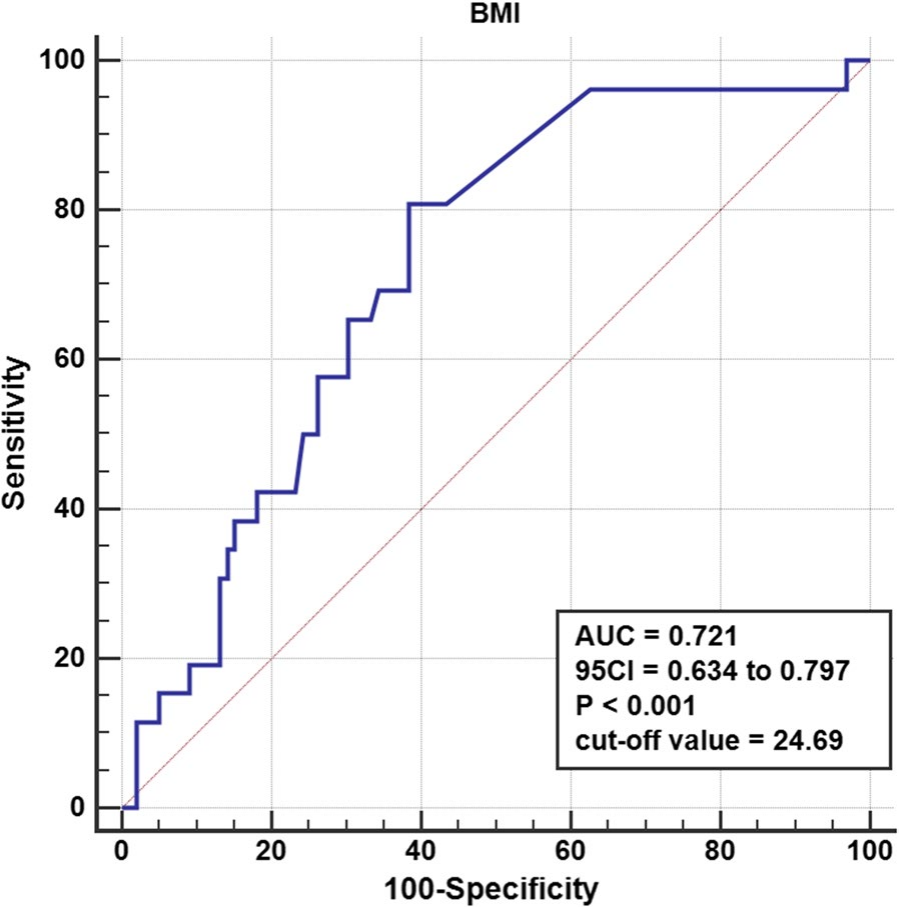
ROC curve for BMI

According to the logistic regression analysis, patients with a higher BMI (*p* = 0.014, OR 1.209, 95% CI (1.040 to 1.406)), a history of smoking (*p* = 0.010, OR 4.185, 95% CI (1.415 to 12.379)), and open fractures (*p* = 0.023, OR 3.714, 95% CI (1.199 to 11.501)) were closely related to the SSI after fasciotomy. Moreover, no protective factors that impact the incidence of infections in these patients were observed in this study (Table 4). Furthermore, Fig. 2 depicts the BMI (*p* < 0.001, AUC area = 0.721, 95% CI (0.634 to 0.797)) as the independent predictor of SSI revealed by the ROC curve analysis, with a cut-off value of 24.69 kg/m^2^.

**Table 4:** Binary logistic regression analysis of variables associated with SSI

Characteristics	OR	95% CI	*p*
BMI	1.209	1.040 to 1.406	0.014*
Open fracture	3.714	1.199 to 11.501	0.023*
Smoking history	4.185	1.415 to 12.379	0.010*
NEU	1.032	0.937 to 1.136	0.524
GLOB	1.094	0.968 to 1.237	0.149
GLU	1.097	0.917 to 1.314	0.312
TCO_2_	1.012	0.862 to 1.189	0.880

## Discussion

ACS is a recognized consequence of both upper and lower limb fractures and is known to occur more often following fractures of the lower extremity, particularly the tibia [3, 27]. ALCS, one of the most severe complications after lower extremity fractures, is a clinical condition caused by trauma or other factors that result in bleeding, edema, or worsened limb perfusion [5]. Delayed treatment may result in irreversible nerve and muscle damage and even death [7]. Therefore, early identification and subsequent fasciotomy are essential to preventing its serious complications. Nowadays, fasciotomy is an effective treatment for ALCS, which can reduce pressure and restore blood flow to the affected compartment, improving the patient's prognosis significantly [5, 28]. It is widely accepted that performing an unnecessary fasciotomy is preferable to failing to recognize compartment syndrome due to the increased risk of myonecrosis and functional loss associated with untreated ALCS. However, increasing fasciotomies may lead to several adverse outcomes, including SSI, bleeding, DVT, and muscle herniation [9–11].

SSI, one of the most frequent complications after surgery, can cause sepsis, reduce the viability of the injured limb, and then lead to widespread organ failure, as well as account for 34.3% of all adverse events associated with surgery [16, 17]. Deformities and pains caused by infections will significantly affect patients' quality of life. Due to SSI's high prevalence and poor prognosis, we believe it is essential to investigate its risk factors and implement preventative measures.

As far as we know, this is the first study focusing on risk factors that might have affected the rate of SSI in patients with ALCS who had experienced a fasciotomy. In the present research, the SSI rate was 20.8% (26 of 125). Based on our results, several predictors of SSI were identified using univariate analysis, including BMI, NEU, GLOB, GLU, TCO_2_, patients with open fractures, and patients with a smoking history. According to logistic regression analysis, BMI levels, open fractures, and smoking history were associated with an elevated risk of SSI. ROC curve analysis revealed that the cut-off value for BMI to predict SSI was 24.69 kg/m^2^. In addition, the most accurate diagnosis was made when BMI, smoking history, and open fractures were all taken together.

In our research, patients with open fractures had an increased incidence of SSI. We also observed that patients with open fractures had a 3.174-fold increased incidence of SSI compared to those with closed fractures. Previously, we thought that the pressure inside the fascial compartment seemed to be released due to the appearance of skin cracks, and therefore, the risk of ALCS was reduced. However, recent retrospective studies suggested that a skin laceration would not reduce the pressure inside the compartments. Obey et al. even discovered that open fractures were a risk factor for ACS [29]. A meta-analysis reviewed 51 articles covering 2980 open and 7893 closed fractures [30]. They discovered that infection rates were 4.2% for closed fractures and 10.6% for open ones. In addition, Ryan et al. and Li et al. all believed that open fractures represented an independent risk factor for SSI [31, 32], which was consistent with our study. In their research, when compared to closed fractures, open fractures increased the prevalence of SSI by roughly 4.53-fold and 1.99-fold, respectively. The increased infection incidence in patients with open fractures may result from damage to the adjacent soft tissues and the entry of environmental contaminants that may interact with the fracture site. Furthermore, to maintain a healthy soft-tissue envelope, patients may have been subjected to repeated debridement; as a result, preoperative waiting time and surgery time would inevitably be extended. In a retrospective study, Abdo Bachoura et al. found that multiple surgical procedures doubled the incidence of surgical site infection [33]. Our findings demonstrated that open fractures continued to pose significant challenges for physicians. Therefore, improving the level of care for patients with open injuries, closely observing the changes in their condition, and the rational application of antibiotics are crucial measures to reduce SSI.

Smoking is a well-known risk factor for SSI after tibial plateau fracture surgery [31] and general spine surgery [34]. However, no research has been able to establish an association between smoking and SSI after fasciotomy. This is the first study to show that smoking is a risk factor for SSI following fasciotomy (OR 4.185). Our study found that smoking history was an independent risk factor for SSI after fasciotomy. Compared to non-smokers, the infection incidence among smokers remained very high, and our results demonstrated that smoking increased the risk of SSI by up to 4.185-fold. Blair et al. [35] found that the influence of smoking on nonunion and infection following fasciotomy cannot be ignored. Li et al. [31] also demonstrated that smoking was an independent predictor of SSI. In a retrospective database review, Ryan et al. [32] found that smoking was associated with elevated infection rates in patients with upper extremity fractures. All of the above studies were consistent with our results. This may be because smoking, even in small amounts, may significantly impact a patient's prognosis via various mechanisms, including vasoconstriction, local tissue hypoxia, and compromising the reparative processes of wound healing and neutrophil defense against microorganisms. Quitting smoking helped reduce the incidence of SSI prior to surgery. Thomsen et al. found that patients who gave up smoking before surgery may halved their risk of postoperative complications compared to those who smoked [36]. Therefore, at the time of hospitalization, surgeons should proactively inform patients about the advantages of quitting smoking and encourage them to take action to quit tobacco.

A higher BMI is becoming a typical comorbidity for patients requiring orthopedic surgery, which significantly affects healthcare system resources. In our study, the level of BMI was significantly higher in the SSI group compared with the non-SSI group. It has been previously shown in the orthopedic trauma literature that higher BMI levels are associated with higher infection rates [37, 38]. In a retrospective multicenter study, Liu et al. [39] found that BMI was independently related to SSI after geriatric hip fracture surgery. In a meta-analysis, Abdallah et al. [40] discovered that a higher BMI was associated with an increased risk of SSI following spine surgery. The above findings were in line with our study. Higher BMI patients had thicker subcutaneous fat, necessitating higher retraction pressures and increasing the likelihood of dead space formation at wound closure [41]. These factors could result in increased tissue necrosis and inadequate vascular perfusion, which inhibit neutrophils' oxidative killing of bacteria and cause wound infection [42]. However, Brophy et al. [43] found that the level of BMI was not connected with the incidence of infection after anterior cruciate ligament (ACL) replacement, which was inconsistent with our study. This may be related to the fact that the subcutaneous fat around the knee joint was minimal and hence did not exhibit substantial alterations with changes in BMI. Furthermore, the severity of the ACL injury and the incision length were much less than those of ALCS, resulting in a lower chance of infection. As a result, a referral to a nutritionist, bariatric surgeon, or exercise counselor may be necessary prior to fasciotomy.

NEU was generally considered an SSI risk factor [22, 44, 45]. In this study's univariate analysis, the difference in NEU between the SSI and non-SSI groups was statistically significant; however, the multivariate analysis failed to identify NEU as an SSI predictor. This was probably related to the small number of ALCS patients who had fasciotomy in these two hospitals during the last decade. Besides, in the univariate analysis, we discovered that the SSI group had a higher level of GLU than the non-SSI group. Cheadle et al. also found that diabetes was an independent risk factor for SSI. Regarding diabetes, however, we found no difference between the two groups [46]. Although there was a difference in GLU levels between the groups, there was no change in the prevalence of diabetes, which might be because ALCS patients are much younger than participants in earlier studies. Patients who are younger have a lesser likelihood of getting serious health conditions such as diabetes and cardiovascular disease, which were considered risk factors for SSI in the previous research but did not manifest similarly in ours [20].

Despite the fact that this is the first study to investigate SSI risk factors in ALCS patients following fasciotomies, several limitations should be acknowledged. First, since this research was retrospective, certain potential variables that may be connected to SSI, such as types of antibiotics, were only partially available. Second, we were unable to include every confounding factor, as with any other multivariate study, and residual confounding still presents a problem. Third, patient-specific variables (such as smoking, drinking, and medical comorbidities) were mostly based on the patient's self-report and were only as reliable as the patient's understanding of their medical issues. Fourth, the sample size of this study was relatively small. A larger sample size and clinical study were required. Fifth, even though all patients were diagnosed with ACS, not every patient received compartment measurements to obtain a more objective diagnosis.

In conclusion, our study revealed that BMI, smoking history, and open fractures were independent predictors of SSI. The cut-off value for BMI in the ROC curve to predict SSI was discovered to be 24.69 kg/m^2^. Given the findings of this study, more studies using larger sample sizes are required. Future studies might potentially focus on the association between SSI rates and other factors, such as operating time. Our findings allowed us to provide an individualized assessment of the risk of SSI in ALCS patients after fasciotomy, allowing for prompt targeted therapy.

## Data Availability

Yes.

## Abbreviations

ALCS: Acute leg compartment syndrome
ACS: Acute compartment syndrome
ASA: American Society of Anesthesiologists
ROC: Receiver operating characteristic curve
AUC: Area under the curve
BMI: Body mass index
ACL: Anterior cruciate ligament
BAS: Basophil
EOS: Eosinophil
HCT: Hematocrit
HGB: Hemoglobin
IMM: Immature
LYM: Lymphocyte
MON: Monocyte
MPV: Mean platelet volume
NEU: Neutrophil
PLT: Platelet
RBC: Red blood cell
WBC: White blood cell
ALB: Albumin
ALP: Alkaline phosphatase
AST: Aspartate aminotransferase
ALT: Alanine transaminase
GLOB: Globulin
Ca: Calcium
CHE: Cholinesterase
CK: Creatine kinase
CREA: Creatinine
DBIL: Direct bilirubin
GLU: Glucose
LDH: Lactic dehydrogenase
TG: Triglyceride
TC: Total cholesterol
TCO_2_: Total carbon dioxide
TP: Total protein
UREA: Ureophil
UA: Uric acid
